# Effect of Cold- and Hot-Break Heat Treatments on the Physicochemical Characteristics of Currant Tomato (*Solanum pimpinellifolium*) Pulp and Paste

**DOI:** 10.3390/foods11121730

**Published:** 2022-06-13

**Authors:** Kandi Sridhar, Hilal A. Makroo, Brijesh Srivastava

**Affiliations:** 1 Department of Food Engineering & Technology, Tezpur University, Tezpur 784 028, Assam, India or sridhar.kandi@agrocampus-ouest.fr (K.S.); or; 2 UMR1253, Science et Technologie du Lait et de l’œuf, INRAE, L’Institut Agro Rennes-Angers, 65 Rue de Saint Brieuc, F-35042 Rennes, France; 3 Department of Food Technology, Islamic University of Science and Technology, Awantipora 192 122, Jammu & Kashmir, India

**Keywords:** underutilized wild species, mathematical modeling, lycopene, viscosity, thermal processing, color

## Abstract

Currant tomato (*Solanum pimpinellifolium*), an underutilized wild species of modern tomato, was investigated to determine the physicochemical properties and understand the effect of cold- and hot-break heat treatments on physicochemical characteristics. Moreover, a new Arrhenius-type equation was used to model the temperature-dependent viscosity of currant tomato pulp and paste. The currant tomato’s porosity, surface area, and lycopene content were 40.96 ± 0.84%, 663.86 ± 65.09 mm^2^, and 9.79 ± 1.88 mg/100 g, respectively. Cold- and hot-break heat treatments had a significant (*p* < 0.05) effect on tomato pulp and paste color change (0.09 to 0.26; 0.19 to 1.96), viscosity (0.06 to 0.02 Pa.s; 0.85 to 0.37 Pa.s), and lycopene content (9.70 to 9.07 mg/100 g; 9.60 to 9.37 mg/100 g), respectively. An Arrhenius-type equation described the temperature-dependent viscosity of currant tomato pulp and paste with activation energy (E_a_) ranging from 7.54 to 11.72 kJ/mol and 8.62 to 8.97 kJ/mol, respectively. Principal component analysis (PCA) revealed a total of variance 99.93% in tomato pulp and paste as affected by the cold- and hot-break heat treatments. Overall, the findings may provide knowledge for design graders and process optimization to develop currant tomato-based products.

## 1. Introduction

Diets rich in fruits and vegetables have received much consumer attention due to their health-promoting properties and therefore play a vital role in human nutrition and food security. The domestication of major plant species, such as rice, wheat, and maize, provides almost 60% of total energy intake [1]. However, over the years, this approach has been dramatically narrowed, and other wild, semi-wild, and domesticated fruits and vegetable species are entirely neglected and underutilized. Underutilized crops represent a critical source of improved food production and a stable food supply for the projected human population. Moreover, many underutilized crops are considered sources of disease resistance, early maturation, resistance to drought, and soil erosion tolerance [2].

Currant tomato (*Solanum pimpinellifolium*), an underutilized wild species of modern tomato, is native to Western South America and grown (0.50 to 1 m in height) in well-drained and moist soils, with quite large bunches [3]. A study identified many disease-resistant genes in currant tomato varieties and good sources of lycopene with high antioxidant traits compared to commercial *Solanum lycopersicon* L. cultivars [4,5]. Currant tomato is an underutilized or undervalued fruit or vegetable crop but an important source of nutrient and bioactive compounds with beneficial properties [6]. A study by Delgadillo-Díaz et al. [7] investigated the physicochemical characteristics and biological activity of *S. lycopersicum* L. and *S. pimpinellifolium* L. in different cropping systems. Similarly, Bojarska et al. [8] investigated the physicochemical properties of different tomato cultivars, including small-sized tomatoes and concluded that the lower hardness and high biological activity of small-fruited tomatoes compared to commercial tomatoes. This indicates that small-sized tomatoes are an attractive source of health-promoting properties. One way to increase the utilization of currant tomatoes is by conducting extensive research regarding the physicochemical properties and its utilization as a raw material for industrial processing and manufacturing of currant tomato-based products. This signifies an additional option for producing lycopene-rich products and further increases food production by value addition and food sustainability. Generally, the physical properties of fruit are an important parameter for designing and modelling processing operations to create a specific model to obtain a high-standardized raw material for stabilizing commercial tomato-based products in the consumer market [9].

Generally, fresh tomato has a short storage life under ambient storage conditions, and thus tomato is processed in the form of pulp, paste, sauce, soup, and puree. Therefore, tomato processing industries consider tomato pulp or paste as the primary quality parameter of the final food product [10]. For example, tomato pulp or paste quality depends on processing conditions, such as temperature, which further influence the physicochemical characteristics [11]. Another study showed the inactivation of pectin-degrading enzymes (i.e., pectin methylesterase and polygalacturonase) in tomatoes through cold- and hot-break heat treatments improves the quality of the end product [12]. However, although these studies have demonstrated the effectiveness of industrial processes on the quality attributes of commercial/modern tomatoes, too little scientific attention has been devoted to the utilization of currant tomatoes and their high-quality end products. We hypothesize that a better understanding of physicochemical and processing methods could provide the basis to develop high-quality currant tomato-derived commercial products at an industrial scale.

Therefore, this study was aimed to determine the physicochemical properties (i.e., linear dimensions, seed and skin weights, juice yield, bulk and true densities, porosity, geometric mean diameter, surface area, sphericity, moisture, pH, total soluble solids, lycopene, and ascorbic acid) of currant tomato and to understand the effect of cold- and hot-break heat treatments on the physicochemical characteristics of currant tomato pulp and paste. Moreover, the change in viscosity of tomato pulp and paste the temperature was modelled by a new Arrhenius approach. This study contributes to the design and handling of currant tomato processing equipment. The findings from modelling and heat treatment can be used to standardize process parameters to maintain the high yield and quality of currant tomato-based processed products.

## 2. Materials and Methods

### 2.1. Sample Collection

**Figure 1:**
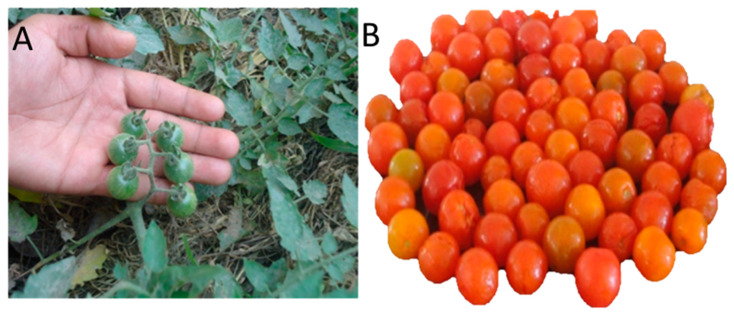
Pictorial representation of currant tomato fruits. Fresh unripe tomato fruits along with twiggy bushes (**A**) and fully ripened fresh currant tomato fruits (**B**).

Currant tomatoes (4 kg) were purchased from the Solmara local market (26° 41′ 25.10″ N and 92° 48′ 2.10″ E), Napaam, Assam, India during January, 2015. Unblemished and mature fresh samples were selected based on visual observation of degree of maturity, size, and color without any mechanical and/or pathological injuries (Figure 1). All the samples were transferred into low-density polyethylene (LDPE) food-grade sampling bags (250 × 200 mm) and then transported to the Department of Food Engineering & Technology, Tezpur University, Assam, India, for further experimental analysis (no longer than two days).

### 2.2. Physicochemical Properties

Fresh fruit samples were thoroughly washed with tap water followed by rinsing with double-distilled water to remove all soil residues. Then, the fruit surface was blotted to dry with a bibulous paper and immediately analyzed to determine physicochemical properties.

The linear dimensions, such as major diameter (length), intermediate diameter (width), and minor diameter (thickness), were measured using a Vernier caliper with a sensitivity of ±0.02 mm. Skin, seed, and juice weights were measured using an electronic balance with an accuracy of ±0.01 g. The water displacement method was used to determine the volume of samples (*n* = 10) [13]. Briefly, randomly selected fruit samples were placed in a measuring cylinder containing a known water volume. The weight of water displaced by the sample was recorded, and the volume was calculated according to Equation (1).

(1) $$\mathrm{Volume}\;\left({\mathrm{cm}}^{3}\right)=\left[\frac{\mathrm{Weight}\;\mathrm{of}\;\mathrm{displaced}\;\mathrm{water}\;\left(W\right)}{\mathrm{Weight}\;\mathrm{density}\;\mathrm{of}\;\mathrm{water}\;\left(\rho \right)}\right]$$

Bulk density (ρ_b_) was measured using the mass to volume relationship according to Li et al. [14], as shown in Equation (2). In contrast, true density (ρ_t_) was calculated according to the liquid displacement method [15]. The porosity (Ɛ) of the samples was determined from bulk and true densities using the relationship according to Equation (3).

(2) $$\mathrm{Bulk}\;\mathrm{density}\;\left(g/{\mathrm{cm}}^{3}\right)=\left[\frac{\mathrm{Weight}\;\mathrm{of}\;\mathrm{sample}}{\mathrm{Total}\;\mathrm{volume}\;\mathrm{of}\;\mathrm{sample}}\right]$$

(3) $$\mathrm{Porosity}\;\left(Ɛ\right)=\left[\left(\frac{\rho_{t}-\rho_{b}}{\rho_{t}}\right)\times 100\right]\;\;$$

where ρ_b_ = bulk density and ρ_t_ = true density.

Geometric mean diameter (GMD), surface area (S), and sphericity (φ) were calculated according to Equations (4)–(6):

(4) $$\mathrm{Geometric}\;\mathrm{mean}\;\mathrm{diameter}\;\left(\mathrm{mm}\right)=\left[\sqrt[{3}]{A\times B\times C}\right]$$

(5) $$\mathrm{Surface}\;\mathrm{area}\;\left({\mathrm{mm}}^{2}\right)=\left[\pi \;{\left(\mathrm{GMD}\right)}^{2}\right]$$

(6) $$\mathrm{Sphericity}\;\left(\%\right)=\left[\left(\frac{\mathrm{GMD}}{A}\right)\times 100\right]$$

where A = major diameter (length), B = intermediate diameter (width), and C = minor diameter (thickness).

Moisture content was analyzed according to the Association of Official Analytical Chemists [16], and the results were expressed as %. The pH was determined by a digital pH-meter (pH 510, Eutech Instruments Pte Ltd., Singapore) using 5 mL of sample and calibrated with acidic and neutral buffer solutions (pH 4.0 and 7.0). Total soluble solids of the samples were analyzed by a traditional hand-held refractometer (Erma Inc., Tokyo, Japan), and the results were expressed as °Brix.

Lycopene content was determined by the method proposed by Fish et al. [17]. Briefly, samples (5 g) were thoroughly mixed with an acetonic solution of butylated hydroxytoluene (0.05%, 5 mL), ethanol (95%, 5 mL), and hexane (10 mL) over a shaker (Orbitek^®^ LT, Chennai, India) for 15 min at 180 rpm and 4 °C. After shaking, distilled water (3 mL) was added to the reaction mixture and then it was kept for shaking at 180 rpm and 4 °C for 5 min. The sample was allowed to separate into phases at ambient temperature, and the absorbance of the hexane organic phase was measured at 503 nm using a UV/Vis spectrophotometer (Shimadzu 1700, Tokyo, Japan). Lycopene content of the sample was calculated using a molar extinction coefficient and a specific multiplication factor (31.20) according to Equation (7) and expressed as mg/100 g.

(7) $$\mathrm{Lycopene}\;\mathrm{content}\;\left(\mathrm{mg}/100\;g\right)=\left[\left(\frac{31.20\times \mathrm{absorbance}}{\mathrm{Weight}\;\mathrm{of}\;\mathrm{sample}}\right)\right]$$

Ascorbic acid content was measured according to the method described by Makroo et al. [10]. Briefly, a crushed sample (5 g) was diluted with distilled water to make the total volume 100 mL, and then metaphosphoric acid (20%, 25 µL) was added. The aliquot (10 mL) was titrated against 2, 6 dichloroindophenols (0.05%, *w*/*v*) until it turned to faint pink color for 20 s. Ascorbic acid standardization was carried out using the AOAC method and was calculated as shown in Equation (8). Ascorbic acid content was expressed as mg/100 g.

(8) $$\;\mathrm{Ascorbic}\;\mathrm{acid}\;\left(\frac{\mathrm{mg}}{100\;g}\right)=\left[\left(\frac{\mathrm{Titer}\;\mathrm{value}\;\times \;\mathrm{Dye}\;\mathrm{factor}\;\times \mathrm{Volume}\;\mathrm{made}\;\mathrm{up}\;\times 100}{\mathrm{Amount}\;\mathrm{of}\;\mathrm{aliquot}\;\times \mathrm{sample}\;\mathrm{weight}\;}\right)\right]$$

### 2.3. Cold- and Hot-Break Heat Treatments

Fresh fruit samples were longitudinally cut into four non-identical pieces and then blended in a laboratory-scale mini-blender (Orpat^®^ HHB-107E, Gujarat, India) over 5 min to make smooth pulp (5.50 ± 0.70 °Brix at 24 ± 2 °C). The pulp was preheated to a temperature of 55 and 65 °C for cold-break processing over 2 min and to 75, 85, and 95 °C for hot-break processing over 2 min, respectively [18]. The pulp mixture was sieved through a cheesecloth to remove the skin, seeds, and other solid residues. For tomato paste, freshly prepared pulp samples were concentrated to a viscous paste (25 ± 1 °Brix at 24 ± 2 °C) in a rotary vacuum evaporator (Eyela, NCB-1200, Tokyo, Japan) at 45 °C under controlled pressure. All the samples were cooled to room temperature and stored in sterilized food-grade reclosable pouches (100 × 150 mm) at 4 °C. Samples without cold- and hot-break heat treatments were treated as a control. The samples were coded as T55 and T65 (cold-break) and T75, T85, and T95 (hot-break). All the experiments were conducted in triplicate.

### 2.4. The Physicochemical Characteristics of Currant Tomato Pulp and Paste

#### 2.4.1. Color Analysis

The color of samples was determined by a colorimeter (UltraScan VIS, Hunter Associates Laboratory, Inc., Reston, VA, USA) in terms of CIELAB color parameters, L (light to dark), a (green to red), and b (blue to yellow). The colorimeter was standardized using a black glossy ceramic plate as a reference to the measurements. The total color difference (ΔE) was calculated according to Equation (9).

(9) $$\mathrm{Color}\;\mathrm{difference}\;\left(\Delta E\right)={\left[{\Delta L}^{2}+{\Delta a}^{2}+{\Delta b}^{2}\right]}^{\frac{1}{2}}$$

where ΔL = difference in lightness, Δa = difference in intensity of red color, and Δb = difference in intensity of yellow color.

#### 2.4.2. Apparent Viscosity

Apparent viscosity measurements were determined using a Rapid Visco Analyser (Newport Scientific Pty. Ltd., Warriewood NSW, Australia). Studies by Makroo et al. [12] and Kapoor and Metzger [19] reported the determination of apparent viscosity by a Rapid Visco Analyser. Briefly, samples (25 mL) were subjected to holding temperatures of 30, 45, 60, 75, and 90 °C and a stirring speed of 100 rpm for 3 min after attaining the required temperature. The apparent viscosity of samples was expressed as Pa.s. Lycopene and total soluble solids for pulp and paste were determined according to the above-mentioned methods [12,17]. Total titratable acidity was determined by titration of samples against NaOH (0.10 N) containing phenolphthalein (0.50%) indicator to an endpoint of faint pink color for 1 min. The results were reported based on % citric acid [12].

### 2.5. Modeling of the Temperature-Dependent Apparent Viscosity of Currant Tomato Pulp and Paste

The effect of temperature on the apparent viscosity of pulp and paste is usually expressed by the linear Arrhenius relationship [20], as shown in Equation (10).

(10) $$\ln\;\left(\eta \right)=\ln\left(A_{s}\right)+\frac{E_{a}}{R}\left(\frac{1}{T}\right)$$

where ƞ = apparent viscosity (mPa.s), R (gas constant) = 8.314 J/mol K, E_a_ = activation energy of flow (J/mol), A_s_ = the pre-exponential (entropic) factor of the Arrhenius equation for the liquid system, and T = absolute temperature (K).

**Figure 2:**
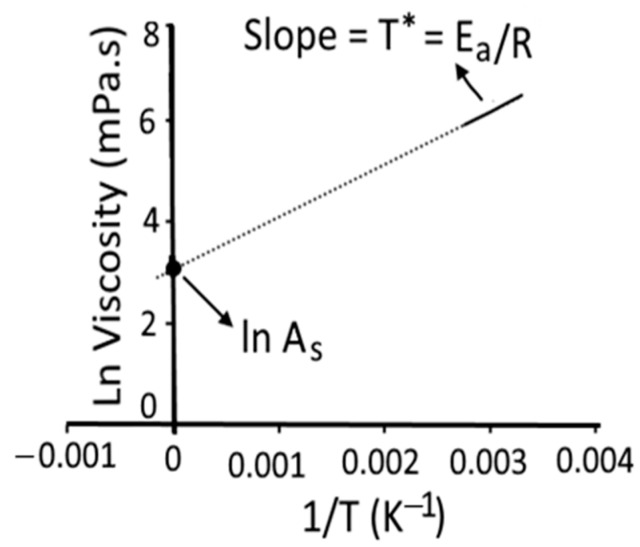
Graphical representation for determination of the Arrhenius parameters: activation energy (E_a_/R), the logarithm of pre-exponential factor (ln A_s_), and Arrhenius activation temperature (T*, K). R = universal gas constant [20]. Figure 2 was reconstructed using experimental data of the present study, which was based on Messaâdi et al. [20] and is available under the Creative Commons Attribution License.

The plot of the logarithm of apparent shear viscosity (ln (η)) vs. reciprocal of absolute temperature (1/T) over different studied temperature ranges was plotted to construct a straight line (R^2^ ≥ 0.98) and intercept [20] as shown in Figure 2. The slope of the straight line is equal to E_a_/R and the intercept on the ordinate is equal to ln A_s_. In addition to E_a_/R and ln A_s_, we determined the Arrhenius temperature (T_A_, K), which was deduced from the intercept according to Messaâdi et al. [20] as shown in Equation (11).

(11) $$T_{A}=\left[\frac{-E_{a}}{R\;\ln\;(A_{s})}\right]$$

The viscosity-temperature dependence can be simplified based on Equations (10) and (11), as shown in Equation (12).

(12) $$\ln\;\left(\eta \right)=\left[\frac{E_{a}}{R}\left(\frac{1}{T}-\frac{1}{T_{A}}\right)\right]$$

For homogenous dimensions, the Arrhenius activation temperature (T*, K) was determined based on Equation (13), as shown in Figure 2.

(13) $$T^{*}=\left[\frac{E_{a}}{R}\right]$$

### 2.6. Principal Component Analysis (PCA)

PCA, an unsupervised multivariate analysis, is used to provide an exploratory grouping of the samples by transferring a set of correlated variables into a new set of linearly uncorrelated variables (i.e., principal components) based on Eigenvalue > 1. We performed the PCA (a Kaiser–Meyer–Olkin value of 0.80, *p* < 0.05) to visualize the differences and similarities between the samples as affected by cold- and hot-break heat treatments using Origin^®^ 2019b version 9.65 (OriginLab Corporation, Northampton, MA, USA). The findings were illustrated by a loading plot containing first two principal components.

### 2.7. Statistical Analysis

The experimental data were reported as the mean ± standard deviation (SD) of ≥three independent determinations. The difference in experimental data was statistically assessed by one-way analysis of variance (ANOVA) using IBM^®^ SPSS^®^ version 16.0 (IBM Ltd., Chicago, IL, USA), and the differences were considered significant at *p* < 0.05 by Duncan’s multiple range test. All graphs were constructed using Microsoft Excel^®^ version 2021 (Microsoft Co., Ltd., Redmond, WA, USA). Bivariate correlation analysis was performed among the defined Arrhenius parameters using IBM^®^ SPSS^®^ version 16.0.

## 3. Results and Discussion

### 3.1. Physicochemical Properties

The physicochemical properties of the currant tomato are presented in Table 1. The average major and minor diameters ranged from 13 to 16.01 mm and 14.02 to 18.03 mm, respectively, with an average intermediate diameter of 14.14 mm at a mean moisture content of 90.86%. The presence of high moisture indicated that the preservation technologies might be required to extend the shelf-life over storage. The fruit contained 40% juice, while 60% contributed to the presence of seeds (34%) and skin (23.30%). The bulk and true density of fruit varied from 0.48 to 0.97 (g/cm^3^) with a porosity of 40.10 to 42.15%. The density of fruits is a fundamental material property and a function of moisture content, which could be used to design materials handling equipment for drying and storage [15]. The high porosity indicated that the drying of currant tomato could be faster with low power requirements. The geometric mean diameter derived from major, minor, and intermediate diameters ranged from 13.67 to 15.93 mm, with a mean value of 14.12 mm.

The surface area and sphericity values varied from 585.91 to 797.12 mm^2^ and 0.98 to 1.04%, respectively. The surface area could determine the wax and packaging material to be applied to the fruit. At the same time, sphericity may be related to the diameter of the fruit and useful for hopper design and pricking machines to handle the fruit [21]. The pH of the fruit was found to be acidic (4.32), with a total soluble solids content ranging from 5 to 7 °Brix. These results agreed with the earlier findings on the pH (4.29) and total soluble solids (5 to 8 °Brix) content of commercial *S. lycopersicon* L. [10,22]. The lower pH was probably due to the presence of organic acids, including citric and maleic acids, that contributed to the tomato’s acidic nature. Lycopene and ascorbic acid contents ranged from 8.21 to 11.45 mg/100 g and 38.15 to 43.06 mg/100 g, respectively, which indicated that the currant tomato is a good source of lycopene and ascorbic acid contents. Similar results were reported for lycopene content in tomato-based products [23]. In brief, the information on the physicochemical properties of any food material may provide knowledge on designing and optimizing process equipment and predicting the behavior of food material. Thus, these findings can assist in developing handling equipment for currant tomatoes.

### 3.2. Effect of Cold- and Hot-Break Heat Treatments on the Physicochemical Characteristics of Currant Tomato Pulp

#### 3.2.1. Color

**Figure 3:**
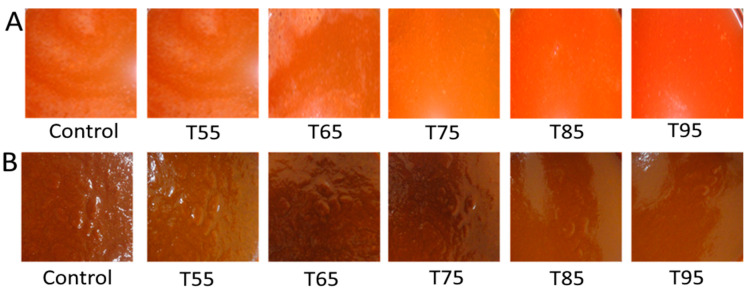
Effect of heat treatments on currant tomato. Pulp (**A**) and paste (**B**). In Figure 3 (**A**,**B**), T55 to T95 represent coded temperature values for cold- and hot-break heat treatments, respectively.

The color parameters of currant tomato pulp as a function of heat treatments indicated a significant difference (*p* < 0.05), as shown in Table 2. The values of the lightness (L), green/red (a), and blue/yellow (b) decreased significantly (*p* < 0.05) at all heat treatments. The variations in L, a, and b values might be due to the consequences of heat treatments that altered the color parameters of tomato pulp [24]. An earlier study by Ganje et al. [25] concluded the variations in color parameters of tomato pulp as affected by thermal processing, which could be related to the degradation of pigments during heat treatments. For example, lycopene in tomatoes may be degraded to *cis* form during heat treatments, resulting in a color change [10]. No significant (*p* > 0.01) change was observed in the a/b value, ranging from 0.99 to 1.04. Application of statistical analysis revealed that heat treatments had a significant (*p* < 0.05) effect on the ∆E of tomato pulp, which further supported the visual appearance of tomato pulp after heat treatments (Figure 3A). Therefore, the findings from this study recommended considering the optimization of heat treatments of tomato juice to maximize the retention of the color attributes of the currant tomato pulp for the consumer’s acceptance.

#### 3.2.2. Apparent Viscosity, Lycopene Content, Total Soluble Solids, and Total Titratable Acidity

**Figure 4:**
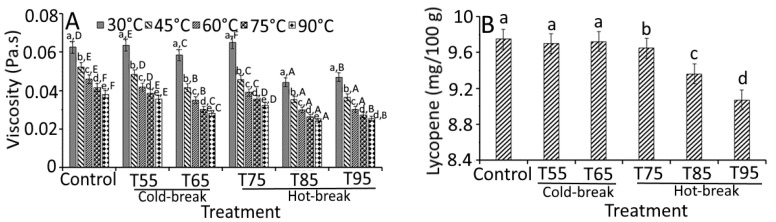

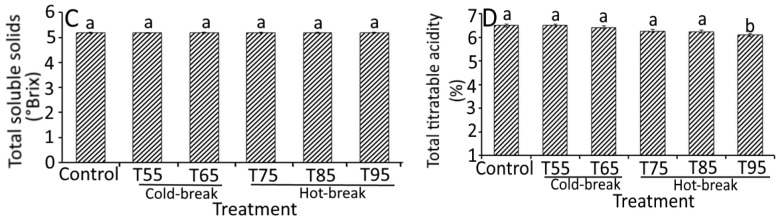
Effect of heat treatments on the quality characteristics of currant tomato pulp. Apparent viscosity (**A**), lycopene (**B**), total soluble solids (**C**), and total titratable acidity (**D**). The error bar represents the standard deviation from the mean of independent triplicate results. Different lowercase letters (a–e) within the treatment indicate a significant difference (*p* < 0.05) based on Duncan’s multiple range test. In Figure 4A, different uppercase letters (A–F) among the holding temperatures indicate a significant difference (*p* < 0.05) based on Duncan’s multiple range test. T55 and T95 represent coded temperature values for cold- and hot-break heat treatments.

Figure 4 shows the apparent viscosity, lycopene content, total soluble solids, and total titratable acidity of tomato pulp as a function of heat treatments. The apparent viscosity of tomato pulp differed significantly (*p* < 0.05) among the heat treatments and followed no definite trend. However, T85 and T95 showed a decreased apparent viscosity (average of 0.03 Pa.s) compared to control (0.05 Pa.s), indicating the effect of heat treatments on currant tomato pulp. The variations in apparent viscosity could be associated with the alignment of the heterogeneous suspended particles that contributed to a strong particle aggregation [18]. Another study by Augusto et al. [26] explained the contribution of particle size in tomato pulp and suggested the homogenization of tomato products to maintain consistency. Heating treatment had a significant impact on the pectin integrity and viscosity of tomato pulp [27]. The heating treatment further showed the conformational changes in pectin solubility, which may change the viscosity of tomato pulp [28]. Another study by Verlent et al. [29] showed the variations in tomato viscosity as affected by heat treatments due to the action of polygalacturonase on pectin, which may be partially active to depolymerization of pectin. Based on the studies mentioned above, we assume that the complexity and heterogeneity of the dispersions may contribute to the variations in the rheological properties of the tomato pulp.

On the other hand, viscosity holding temperatures (30, 45, 60, 75, and 90 °C) showed a significant (*p* < 0.05) effect on the apparent viscosity of tomato pulp (Figure 4A). At 45 °C, heat-treated samples collectively showed a 0.90-fold lower apparent viscosity than the control sample at 30 °C. Moreover, a similar decreased trend was observed for viscosity at different holding temperatures, which was the lowest at 90 °C. At 90 °C, all treated samples demonstrated a 0.95-fold lower apparent viscosity than the control sample (0.04 Pa.s). These findings were likely related to the rise in temperature and heat treatments [18].

A significant difference (*p* < 0.05) in lycopene content and total titratable acidity was observed based on the heat treatments except for the TSS content, where heat treatments showed (Figure 4B–D) insignificant change (*p* > 0.05). Similarly, Boubidi and Boutebba [30] reported the insignificant change in TSS content of tomato products as affected by the change in temperature, which may relate to the short time treatments. For lycopene content, no significant change was observed in T55, T65, and T75 compared to control. However, a slight but significant decrease in lycopene content was observed in T85 (9.36 mg/100 g) and T95 (9.07 mg/100 g). At a higher temperature (130 °C), Miki and Akatsu [31] reported the loss of lycopene content (0.17 mg/100 g), which could be related to the higher degradation of *trans* lycopene to *cis* form [25]. High loss of lycopene at high holding temperature has also been reported due to dissolved air in the pulp that may destroy lycopene [32]. Therefore, the degradation of tomato pulp during thermal processing can be reduced by de-aerating the pulp before thermal treatment and reducing the duration of treatment. The total titratable acidity was decreased significantly from 6.50 (control) to 6.10% (T95), which was found to be 0.40% lower than control. We propose that reducing total titratable acidity could be the consequences of inactivated pectin enzyme activity and solubilization of acids in an aqueous medium. A study by Boubidi and Boutebba [30] highlighted the interdependence of natural acids by heat treatments. Compared to the natural acids, heat treatments significantly affected the parameters like apparent viscosity, lycopene content, and total titratable acidity.

### 3.3. Effect of Cold- and Hot-Break Heat Treatments on the Physicochemical Characteristics of Currant Tomato Paste

#### 3.3.1. Color

The color characteristics of the currant tomato paste are summarized in Table 2. The L value significantly (*p* < 0.05) ranged from 25.54 to 27.58, while the ‘a’ value varied from 8.99 to 9.88 in all heat treatments. Similarly, there was a significant change (*p* < 0.05) in color ‘b’ value after heat treatments, ranging from 5.89 to 6.24. Moreover, the a/b value had an insignificant effect (*p* > 0.05) that ranged from 1.48 to 1.52. As shown in Table 2, heat treatments had a significant decrease in ∆E of currant tomato paste, which varied from 0.19 to 1.96. According to the findings from the color characteristics of currant tomato paste, it can be concluded that the tomato paste followed a similar trend as the color characteristics of the currant tomato pulp. This indicated that the heat treatments had a similar influence on currant tomato pulp and paste color characteristics. Our visual scoring of currant tomato pastes also evidenced the change of color attributes (Figure 3B). Similar findings were documented by Shatta et al. [10] that showed significant changes in color parameters (L, a, b, a/b, and ∆E) of tomato paste after thermal processing. Another study by Ganje et al. [25] highlighted the change in the color characteristics of tomato paste overheat processing, which could be the consequences of lycopene thermal degradation from *trans* to *cis* form and isomerization. Considering the color change characteristics of currant tomato paste, application of heat treatments on tomato products should be carefully monitored to receive high consumer perception on thermally processed currant tomato-based products.

#### 3.3.2. Apparent Viscosity, Lycopene Content, Total Soluble Solids, and Total Titratable Acidity

**Figure 5:**
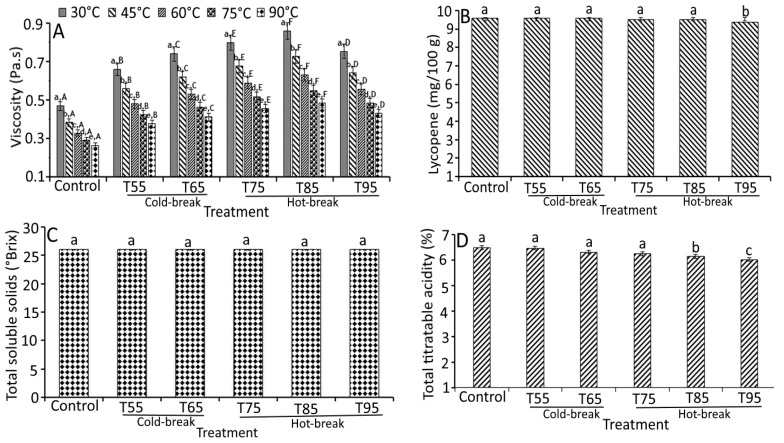
Effect of heat treatments on the quality characteristics of currant tomato paste. Apparent viscosity (**A**), lycopene (**B**), total soluble solids (**C**), and total titratable acidity (**D**). The error bar represents the standard deviation from the mean of independent triplicate results. Different lowercase letters (a–e) within the treatment indicate a significant difference (*p* < 0.05) based on Duncan’s multiple range test. In Figure 5A, different uppercase letters (A–F) among the holding temperatures indicate a significant difference (*p* < 0.05) based on Duncan’s multiple range test. T55 and T95 represent coded temperature values for cold- and hot-break heat treatments.

The apparent viscosity, lycopene, total soluble solids, and total titratable acidity of currant tomato paste as a function of heat treatments are depicted in Figure 5A–D. The findings reported that the apparent viscosity, lycopene, and total titratable acidity of currant tomato paste differed significantly (*p* < 0.05), but an insignificant (*p* > 0.05) change was observed in the total soluble solids content of currant tomato paste (Figure 5). The apparent viscosity values (0.47 to 0.85 Pa.s) of currant tomato paste demonstrated an increased trend for heat treatments (T55 to T85) and then decreased to 0.75 Pa.s (T95). Higher apparent viscosity for currant tomato paste could be ascribed to the solid concentration of the samples, indicating that solids content plays a vital role in the consistency of tomato paste [22]. For heat treatments, the highest apparent viscosity was observed for T85 at a holding temperature of 30 °C (0.85 Pa.s), whereas the lowest was recorded for control (0.26 Pa.s) at a holding temperature of 90 °C (Figure 5A). This indicated that the heat treatments influenced the viscosity of currant tomato paste. For example, T85 reported the maximum apparent viscosity at all holding temperatures (30 to 90 °C) compared to control, T55, T65, and T75. This could be related to the action of pectin enzymes due to their strong intra- and inter-molecular bonding of pectin. At a higher temperature (T95), significantly lower apparent viscosity was observed than at T75 and T85. A possible explanation for this might be related to the breakdown of pectin structural units of the sample. Other researchers have suggested the low viscosity of tomato paste during heat treatment [22].

On the other hand, we compared the influence of holding temperature on apparent viscosity of currant tomato paste (Figure 5A). The apparent viscosity of currant tomato paste significantly decreased (*p* < 0.05) in control and heat-treated samples over the rise in holding temperature from 30 to 90 °C. The heat-treated samples showed a 1.62-fold higher apparent viscosity than the control sample at a holding temperature of 30 °C, which followed a similar tendency at all holding temperatures (45 to 90 °C). These differences can be partially elucidated by the proximity of holding temperature and heat treatments.

Lycopene content and total titratable acidity significantly varied and ranged from 9.75 to 9.07 mg/100 g and 6.5 to 6.10%, respectively, for all treatments. Still, no significant change (*p* > 0.05) was observed in total soluble solids (5.20 °Brix). Similar findings were reported by Kaur et al. [33] that showed a slight variation in lycopene content during different processing methods. At higher heat treatments (T75 to T95), lycopene content was found to be lower (~9.36 mg/100 g) than in control (9.75 mg/100 g), indicating that the lycopene was stable at a lower temperature (T55 and T65) and then degraded at higher temperature (T75 to T95). This was similar to the lycopene degradation findings of Shi et al. [34], where lycopene was stable at lower temperatures. Then, a significant decrease was observed over a temperature rise. The maximum decrease in total titratable acidity of the currant tomato paste was obtained at T95, which was 0.82-fold lower than the control (6.50%). This may be due to the precipitation of salts existing in the paste [35]. Based on our findings, it can be concluded that the heat treatments may influence the viscosity and quality attributes of currant tomato paste. Hence, based on requirements, heat treatments and holding temperature may be altered for a subsequent application in the development of currant tomato products.

### 3.4. Modeling of the Temperature-Dependent Apparent Viscosity of Currant Tomato Pulp and Paste

**Figure 6:**
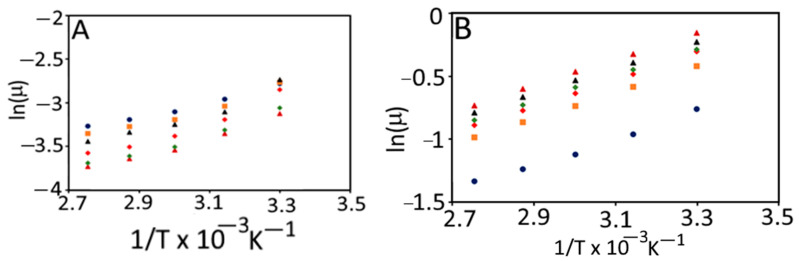
Arrhenius plot for the viscosity of currant tomato pulp (**A**) and paste (**B**). Control (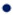), T55 (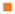), T65 (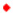), T75 (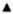), T85 (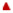), and T95 (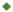). T55 and T95 represent coded temperature values for cold- and hot-break heat treatments.

In general, the effect of temperature on the apparent viscosity of currant tomato pulp and paste is explained according to the Arrhenius equation (Equation (10)). The Arrhenius model constants were evaluated in terms of natural log (ln) of viscosity vs. inverse of absolute temperature (1/T, K^−1^) for pulp and paste (Figure 6). The activation energy values for pulp and paste were observed in the range of 7.54 to 11.72 kJ/mol and 8.62 to 8.97 kJ/mol, respectively. The coefficient of determination (R^2^) for pulp and paste was >0.98, which explained the strong linear relationship between inverse of temperature (1/T) and apparent viscosity of pulp and paste. These findings agreed with earlier findings, which reported activation energy values from 8.60 to 14.08 kJ/mol for tomato concentrates [36].

The Arrhenius temperature (T_A_, K) and the Arrhenius activation temperature (T*, K) for pulp and paste are shown in Table 3. The T_A_ and T^*^ showed interdependence and ranged from 313.49 to 6106 K and 907.77 to 1410.75 K for both pulp and paste, respectively. We further performed the correlation analysis among the defined Arrhenius parameters (T_A_, ln A_s_, and E_a_) for currant tomato pulp and paste (Table 4). ln A_s_ and E_a_ exhibited a negative correlation with T_A_, while ln A_s_ showed a positively weak correlation with E_a_ for tomato pulp. The similar correlation trend was reported for paste, where T_A_ showed a strong negative correlation (−0.90) with ln A_s_; however, T_A_ for paste demonstrated a strong positive correlation (0.79) with E_a_ compared to pulp. This indicated the interdependence of Arrhenius parameters, which may highly influence the determination of E_a_ for food products. A study by Messaâdi et al. [20] reported the linear and non-linear correlations among the Arrhenius parameters. Thus, these results concluded that the currant tomato pulp and paste obey the linear Arrhenius behavior. An Arrhenius equation could describe the temperature dependence of viscosity of currant tomato pulp and paste. Hence, we recommend using a new Arrhenius-type equation with two and/or three parameters and thus useful for determining the nature of fluids.

### 3.5. Principal Component Analysis (PCA)

**Figure 7:**
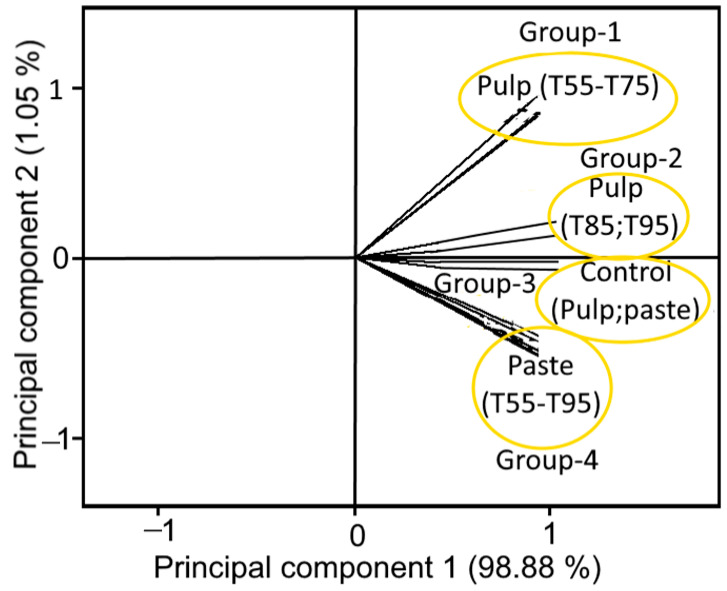
Loading plot of the first two principal components of tomato samples as affected by cold- and hot-break heat treatments. T55 and T95 represent coded temperature values for cold- and hot-break heat treatments.

The PCA of tomato samples as affected by cold- and hot-break heat treatments resulted a total variance of 99.93%, in which first two principal components (PCs 1 and 2) represented a major variance of 98.88% and 1.05%, respectively with an Eigenvalue >1, indicating close interdependence of the samples. Figure 7 illustrates the clear separation of samples within the quadrants 1 and 4 (Q1 and Q4), indicating the effect of cold- and hot-break heat treatments that had different influences on tomato pulp and paste. Moreover, both pulp and paste samples formed well-separated groups as a function of cold- and hot-break heat treatments. For example, pulp samples treated with T55, T65, and T75 formed a group 1, while group 2 was formed by the pulp samples treated with T85 and T95. However, paste samples treated with cold- and hot-break heat treatments (T55, T65, T75, T85, and T95) collectively formed group 4 (Figure 7). Likewise, untreated samples (control pulp and paste) were clearly separated as group 3. The formation of different groups could be related to the similarities (due to less angle) among the samples treated with cold- and hot-break heat treatments. Thus, samples with less angle showed a high similarity and were close to each other among the samples. Based on these observations, the findings concluded that the tomato pulp and paste characteristics might have been influenced by the cold- and hot-break heat treatments and could be considered while preparing heat-treated tomato-based processed products.

## 4. Conclusions

The results showed the physicochemical properties of currant tomato pulp and paste. Currant tomato pulp and paste were significantly affected by the heat treatments. Color, lycopene content, and viscosity of tomato pulp and paste were significantly influenced by the cold- and hot-break heat treatments. However, total soluble solids, and total titratable acidity showed no significant differences after the cold- and hot-break heat treatments. The activation energy values ranged from 7.54 to 11.72 kJ/mol and 8.62 to 8.97 kJ/mol, respectively, for pulp and paste. The results further indicated that the linear Arrhenius-type equation successfully described the temperature-dependent apparent viscosity of currant tomato pulp and paste. PCA analysis revealed the differences in tomato pulp and paste characteristics (a total variance of 99.93%) as affected by the cold- and hot-break heat treatments. This is one of the first attempts to provide the basic physicochemical information on currant tomatoes that could be used in the design of currant tomatoes cleaning, handling, and separation machines. Data from heat treatments and modeling can be used to control and optimize process parameters in developing high-quality currant tomato-based food products.

## Figures and Tables

**Table 1:** Descriptive statistics for the physicochemical characteristics of currant tomato ^1^.

Parameter	Minimum	Maximum	Mean	SD
Major diameter (mm)	13	16.01	14.31	0.82
Intermediate diameter (mm)	13.01	17.01	14.14	1.08
Minor diameter (mm)	14.02	18.03	14.92	1.22
Skin weight (%)	22.05	25.42	23.30	0.02
Seed weight (%)	32.00	35.25	34.00	0.01
Juice (%)	38.96	42.78	40.00	0.01
Bulk density (g/cm^3^)	0.48	0.59	0.54	0.04
True density (g/cm^3^)	0.88	0.97	0.92	0.03
Porosity (%)	40.10	42.15	40.96	0.84
Geometric mean diameter (mm)	13.67	15.93	14.12	0.70
Surface area (mm^2^)	585.91	797.12	663.86	65.09
Sphericity (%)	0.98	1.04	1.01	0.08
Moisture (%)	88.1	93.63	90.86	1.81
pH	4.30	4.60	4.32	0.12
Total soluble solids (°Brix)	5	7	5.50	0.70
Lycopene (mg/100 g)	8.21	11.45	9.79	1.88
Ascorbic acid (mg/100 g)	38.15	43.06	39.93	1.88

^1^ The results were expressed as the mean ± standard deviation (*n* ≥ 3). SD = standard deviation of the mean determined from ≥3 independent determinations.

**Table 2:** Effect of heat treatments on the color parameters of currant tomato pulp and paste ^1^.

Treatment	L	a	b	a/b	∆E
Pulp					
Control	29.45 ± 0.54 ^d^	7.61 ± 1.08 ^e^	7.32 ± 0.62 ^c^	1.04 ± 0.01 ^a^	0.00 ± 0.00 ^a^
T55	29.40 ± 0.97 ^d^	7.53 ± 2.28 ^d^	7.31 ± 0.83 ^c^	1.03 ± 0.05 ^a^	0.09 ± 0.01 ^b^
T65	29.33 ± 1.04 ^d^	7.47 ± 2.11 ^c^	7.34 ± 0.14 ^c^	1.02 ± 0.01 ^a^	0.09 ± 0.01 ^b^
T75	29.11 ± 2.01 ^c^	7.40 ± 0.08 ^c^	7.21 ± 0.22 ^b^	1.03 ± 0.07 ^a^	0.26 ± 0.04 ^c^
T85	28.92 ± 3.33 ^b^	7.27 ± 1.29 ^b^	7.25 ± 0.28 ^b^	1.01 ± 0.02 ^a^	0.23 ± 0.04 ^c^
T95	28.78 ± 0.07 ^a^	7.13 ± 1.58 ^a^	7.17 ± 0.09 ^a^	0.99 ± 0.08 ^a^	0.21 ± 0.05 ^c^
Paste					
Control	25.54 ± 0.71 ^a^	9.88 ± 0.05 ^c^	6.54 ± 0.08 ^e^	1.51 ± 0.01 ^a^	0.00 ± 0.00 ^a^
T55	27.15 ± 0.85 ^c^	8.91 ± 0.03 ^a^	5.97 ± 0.01 ^b^	1.49 ± 0.09 ^a^	1.96 ± 0.09 ^f^
T65	26.94 ± 0.34 ^b^	9.12 ± 0.06 ^a^	6.12 ± 0.09 ^c^	1.49 ± 0.08 ^a^	0.33 ± 0.01 ^d^
T75	26.99 ± 0.68 ^b^	9.27 ± 0.01 ^b^	6.24 ± 0.01 ^d^	1.48 ± 0.05 ^a^	0.19 ± 0.01 ^b^
T85	27.26 ± 0.55 ^c^	9.21 ± 0.04 ^b^	6.19 ± 0.05 ^c^	1.48 ± 0.01 ^a^	0.28 ± 0.01 ^c^
T95	27.58 ± 0.26 ^d^	8.99 ± 0.05 ^a^	5.89 ± 0.06 ^a^	1.52 ± 0.09 ^a^	0.49 ± 0.01 ^e^

^1^ The results were expressed as the mean ± standard deviation (*n* = 3). Mean values within a column with different lowercase superscripts (a–f) were significantly different (*p* < 0.05, Duncan’s multiple range test). T55 and T95 represent coded temperature values for cold- and hot-break heat treatments.

**Table 3:** Arrhenius parameters for temperature-dependent apparent viscosity of currant tomato pulp and paste ^1^.

Treatment	T* (K)	ln A_s_	E_a_ (kJ/mol)	T_A_ (K)
Pulp				
Control	907.77	1.12	7.54	810
T55	1071.83	0.57	8.91	1877
T65	1410.75	0.62	11.72	2264
T75	1242.12	0.01	10.32	1035
T85	1117.59	0.07	9.29	1490
T95	1092.95	0.18	9.08	6106
Paste				
Control	1076.27	2.58	8.94	417.18
T55	1040.19	3.05	8.64	340.61
T65	1079.61	3.03	8.97	355.46
T75	1028.20	3.28	8.54	313.49
T85	1080.03	3.19	8.97	338.53
T95	1030.50	3.20	8.62	324.11

^1^ T* = Arrhenius activation temperature, A_s_ = the pre-exponential (entropic) factor of the Arrhenius equation for the liquid system, E_a_ = activation energy of flow, and T_A_ = Arrhenius temperature. T55 and T95 represent coded temperature values for cold- and hot-break heat treatments.

**Table 4:** Correlation among the defined Arrhenius parameters of currant tomato pulp and paste ^1^.

Sample	Arrhenius Parameters	T_A_	ln A_s_	E_a_
Pulp	T_A_	1	−0.01	−0.29
	ln A_s_		1	0.32
	E_a_			1
Paste	T_A_	1	−0.90 *	0.79
	ln A_s_		1	−0.46
	E_a_			1

^1^ T_A_ = Arrhenius temperature, A_s_ = the pre-exponential (entropic) factor of the Arrhenius equation for the liquid system, and E_a_ = activation energy of the flow. * Correlation is significant at the 0.05 level (2-tailed). T55 and T95 represent coded temperature values for cold- and hot-break heat treatments.

## Data Availability

The data that supports the findings of this study are available within the manuscript.
